# Round the Hospitals

**Published:** 1920-11-13

**Authors:** 


### ROUND THE HOSPITALS.

The King held an Investiture in the Ballroom of Buckingham Palace on November 4, and personally conferred decorations on members of the nursing services as follows:—Bar to the Royal Red Cross.—Miss Edith Holden, T.F.N.S. Royal Red Cross, First Class.—Miss Janet Durward, Miss Adelaide Russell, Miss Nina Stokes, Miss Lilian Wass, and Miss Elizabeth Williams, Q.A.I.M.S.R.; Miss Marion Andrew, Miss Winifred Bickham, Miss Rose Black, Miss Corrina Dent, Miss Lilian Holden, Miss Sarah Lewis, Miss Kathleen Smith, Miss Maud Weale, and Miss Effie White, T.F.N.S.; Mrs. Mary Wisdom, C.N.S.; Miss Rosamund Skipworth, Mrs. Kate Talbot, B.R.C.S.; Mrs. Maud Tennyson-Smith, Mrs. Louise Wilson, V.A.D.

---

The harmony which reigned at the meeting of the College of Nursing on November 4 made it a very pleasant function to attend. The large number of nurses present showed that the interest of members is well maintained, and the excellent remarks made by some who came from a distance, and have not previously been heard, reveals that there are untapped powers of sound, sensible speech to be relied on among the "silent" members. The actual rates of subscription were not for the meeting to decide. It had merely to confer powers on the Council to fix a subscription for all elected after November 20. The proposition that for existing members the subscription should be purely voluntary was never for a moment in doubt. Miss Good was on sure ground when she urged that the amount existing members be asked to subscribe voluntarily should be definitely limited to 5s. The right of appeal granted to members whose name it may be proposed to strike off the Register meets, as the Chairman remarked, "the only criticism of the Articles of Association."

---

When the Queen devoted the surplus funds of Queen Mary's Needlework Guild to the foundation of the maternity home at Hampstead for people with small means she did much to popularise homes of this valuable description. Her Majesty's visit on Saturday to the Wandsworth Borough Maternity Home at Parkhill, Tooting, proved the continued interest she takes in the provision of lying-in accommodation for residents in flats and small houses containing no proper conveniences for a confinement. The Matron, Miss Waring, who had the honour of conducting the Queen over the new institution, is assisted by an excellent staff of nurses, and the highest medical skill is available for the patients. There are twenty-two beds at a payment varying from £1 to 4 guineas a week, and only in exceptional cases will non-paying patients be received. It is a very happy instinct which has induced Wandsworth to carry out this admirable enterprise as a war memorial. The wives of many men who fought in the war will be the first to benefit from it. The Queen's untiring interest in all the thousand-and-one details which go to make success in such homes was helpful and inspiring.

---

The projected home for child victims of venereal disease will meet an urgent need. A powerful appeal has been circulated under the auspices of Princess Christian, Lady St. Helier, the Bishop of London, Dr. Mary Scharlieb, and others, and it is stated a suitable house with excellent grounds is available near London, that two trained nurses are willing to take charge, and that a substantial grant has been promised from the Health authorities. The urgency of the situation may be gauged from the fact that at one hospital in London no fewer than 134 infected children, all under fourteen years of age, attended as out-patients in the course of six months. Twelve little girls between the ages of three and twelve will be at first received and later it is hoped to find accommodation for twenty-five, including some little boys under nine. It is of course not nearly enough, but it is much to make a beginning. The Hon. Treasurer is Mr. R. Davies-Colley, M.Ch., F.R.C.S., 10 Devonshire Place, W. 1.

---

A most successful Café Chantant and Sale of Work was held at the Guild Hall, Gloucester, on November 3 and 4, organised by Mrs. J. O. Roberts, the Mayoress, and a band of loyal helpers. Its object was to provide funds for a much-needed electric lift for the Royal Infirmary. The enthusiasm and liberal support it evoked in the city and county of Gloucester speaks well for the estimation in which the infirmary is held by all classes. The sale was opened the first day by the Duchess of Beaufort, one of the patrons of the hospital, with Sir James Bruton, M.P., in the chair, and the second day by Lady Marling, with Mr. J. O. Roberts, the Mayor, as chairman. The splendid response for donations to the stalls exceeded all expectations. The county produce stall was so liberally supplied that it contributed over £800 to the amount taken. Other stalls were also nobly equipped, and the full sum required for the lift, £1,500, was more than realised, and reached the magnificent sum of £1,900.

---

By a regrettable oversight in criticising lately the meagreness of the nurses' share in the King's College Hospital Report we overlooked the list of Red Cross Honours allotted to the staff, numbering ten First Class and ten Second Class. In the Roll of Honour the names of three sisters appear—Sister Elise Kemp, killed October 20, 1917; Sister Elizabeth Robinson, died of malaria July 14, 1919; Nurse F. Compton, drowned at Basra December 1917. What we should like to see in the next edition of this admirably put together Report is a record of the work accomplished in the nurse training-school, to include the number of entrants, the number of certificated, a list of prizewinners, and some record of the causes of leaving, etc. Such records are not only of great interest, they are stimulating to other schools, and collectively have their value in the history of nursing.

The autumn meeting of the Scottish Matrons' Association took place at the Nurses' Club, 8 Drumsheugh Gardens, on October 30, when the President, Miss Gill, welcomed members to their first meeting in the Edinburgh Nurses' Club. Four new members were elected, and discussion took place on the question of an educational standard for entrants, which had been referred to the Association by the College of Nursing. Other matters under discussion were Unemployment Insurance and the Registration of Nurses. The proceedings terminated with an enjoyable tea in the club.

We are informed that the committee of the General Hospital, Birmingham, have appointed to the vacant post of food supervisor Mrs. E. Lloyd, at present housekeeper at the Highbury V.A.D. Hospital, Birmingham.

Princess Alice's Home at Slough for Disabled Sailors and Soldiers who are suffering from paralysis has just issued its first annual report. It is very interesting to find that in many of these apparently incurable cases remarkable improvement has rewarded the efforts of the staff. Most of the men when admitted had been bedridden for long periods, but few are now unable to be up for part of the day. Some cases, deemed hopeless at first, are now walking, and are regaining confidence and strength daily. Members of the medical and nursing staff are most keen and devoted, and the surprising results should set a new current of hope stirring in many minds. Subscriptions may be sent to H.R.H. Princess Alice, Henry III. Tower, Windsor Castle.

At Witham, as well as at Wakefield, the war memorial is to take the form of a nurses' home, and on October 27 the foundation stone was laid by Lady Rayleigh of the building which is destined, we believe, to prove a great source of alleviation to sufferers in the town. It has been planned to contain a maternity ward for the reception of any case requiring special treatment, and will afford greatly needed increased accommodation for the nurses of the Witham Nurses' Association. The site was presented by Mr. P. E. Laurence, and the building as a whole will cost £2,000.

